# A Case of Humoral Hypercalcemia of Malignancy Secondary to Hepatocellular Carcinoma with Fulminant Clinical Course

**DOI:** 10.51894/001c.8983

**Published:** 2019-07-01

**Authors:** Pamela Castro-Camero, Bumsoo Park, Amit Gupta, Raghavendra Vemulapalli, Maria Shreve

**Affiliations:** 1 Family Medicine Henry Ford Health System; 2 Family Medicine and Urology University of Michigan (MI); 3 Primary Care Aurora Medical Center

**Keywords:** parathyroid-hormone related peptide, hypercalcemia, hepatocellular carcinoma

## Abstract

Hepatocellular carcinoma is one of the most common liver malignancies in the United States. Poor prognosis is associated with paraneoplastic syndromes such as hypercalcemia, hypercholesterolemia, or hypoglycemia. Hypercalcemia as a paraneoplastic syndrome of hepatocellular carcinoma has been rarely reported. We report a mortality case of incidentally diagnosed hepatocellular carcinoma associated with humoral hypercalcemia of malignancy. The patient demonstrated a fulminant disease course with an unexpected fatal outcome within 40 days of initial diagnosis. Our case can suggest importance of early definitive treatment of hepatocellular carcinoma, extremely close monitoring, and aggressive medical treatment when it is associated with humoral hypercalcemia of malignancy.

## INTRODUCTION

Hypercalcemia, or high serum calcium level, in cancer patients is a serious and common finding in squamous cell carcinoma (a malignant tumor arising from squamous epithelium such as skin), multiple myeloma (a malignant tumor of the plasma cells), as well as renal, bladder, breast or ovarian carcinomas.^1^ The pathophysiology through which hypercalcemia may occur in malignancy is via three mechanisms: 1) tumor secretion of parathyroid hormone-related protein (PTHrP), also known as humoral hypercalcemia of malignancy (HHM); 2) release of cytokines after metastases to bone tissue; and 3) tumor production of calcitriol.^2^

While the association between hypercalcemia and liver malignancy has been documented, its incidence is rare, having been reported as 4-8%.^3^ Approximately 40 case reports regarding the association of hypercalcemia and hepatocellular carcinoma (HCC) have been published since the first 1963 report.^4^ The presence of paraneoplastic syndromes (clinical syndromes that are the consequence of malignant disease) such as hypercholesterolemia, hypoglycemia, and hypercalcemia in HCC are known to be a poor prognostic factors.^5^ In addition, many of the case reports have shown that definitive therapy for HCC could control hypercalcemia.^5,6^

In this paper, the authors report a case of hepatocellular carcinoma in a patient who presented with non-specific symptoms and was later found to have hypercalcemia due to paraneoplastic secretion of PTHrP from the HCC. The patient’s hypercalcemia failed to be properly controlled resulting in a fulminant and fatal outcome.

## CASE REPORT

A female in their late 60’s with a history of daily alcohol use presented to the emergency department with new onset confusion, generalized weakness, and abdominal pain. Physical examination was negative for any focal neurological deficit except altered mentation. Blood chemistry showed corrected total serum calcium level of 12.6 mg/dL, aspartate aminotransferase (AST) of 129 IU/L, total bilirubin of 2.3 mg/dL, direct bilirubin of 1.0 mg/dL, and serum ammonia of 56 umol/L.

Her Hepatitis C antibody was positive. Both computed tomography (CT) of the head and magnetic resonance imaging of the brain were negative for acute stroke. Her altered mentation was attributed by the authors to a toxic-metabolic etiology. She was treated with intravenous (IV) 0.9% sodium chloride infusion, lactulose, and rifaximin, which did not improve her mentation.

A CT of the liver showed a 10 x 6 cm-sized right hepatic mass demonstrating arterial hypervascularity and venous washout features of HCC with a few satellite masses in the setting of cirrhosis (Figure 1A). A right main portal vein tumor thrombus was also seen (Figure 1B).

**Figure 1A.**
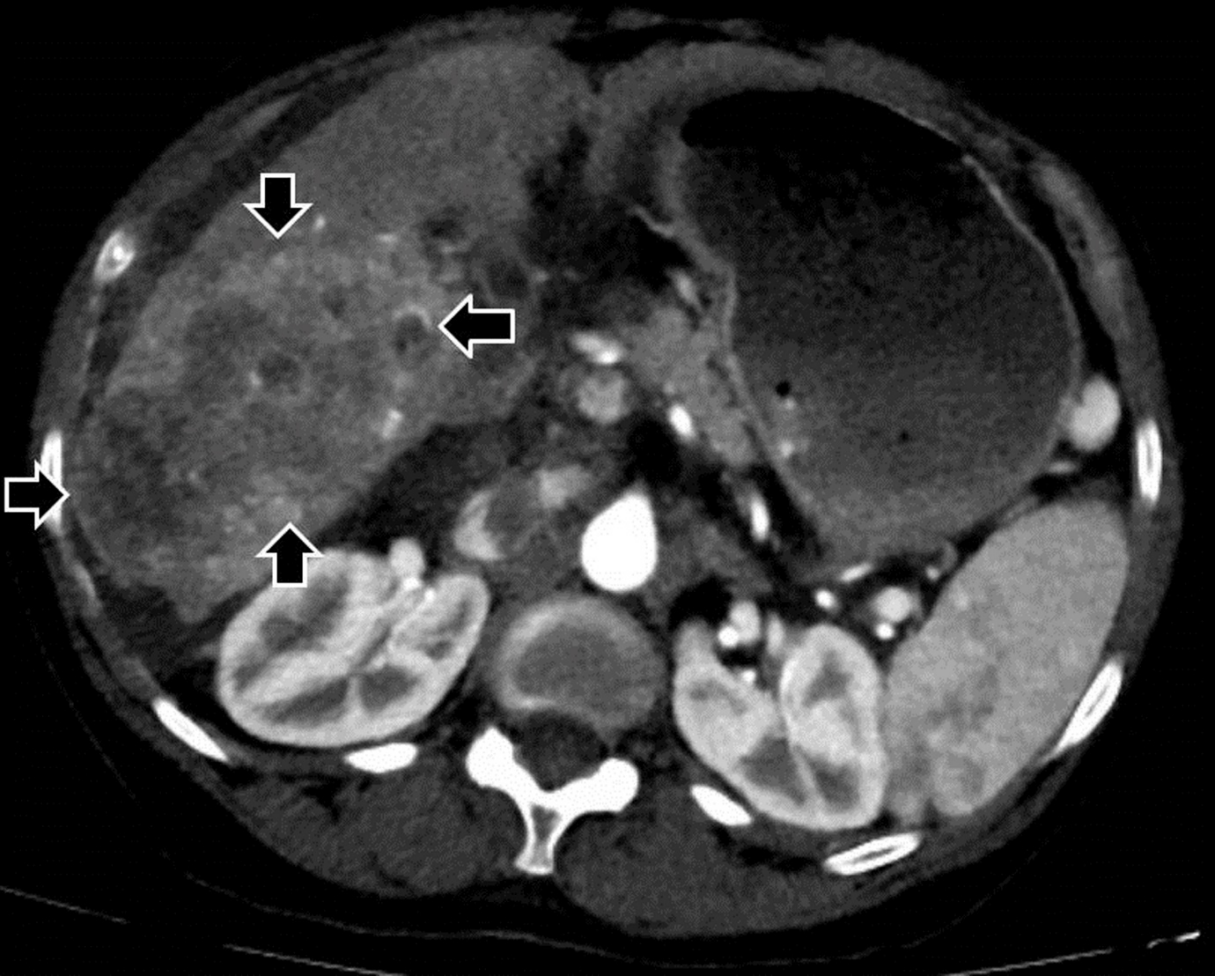
Computed tomography image of the liver with contrast showing a large, about 10 x 6 cm-sized right hepatic mass (black bold arrows).

**Figure 1B.**
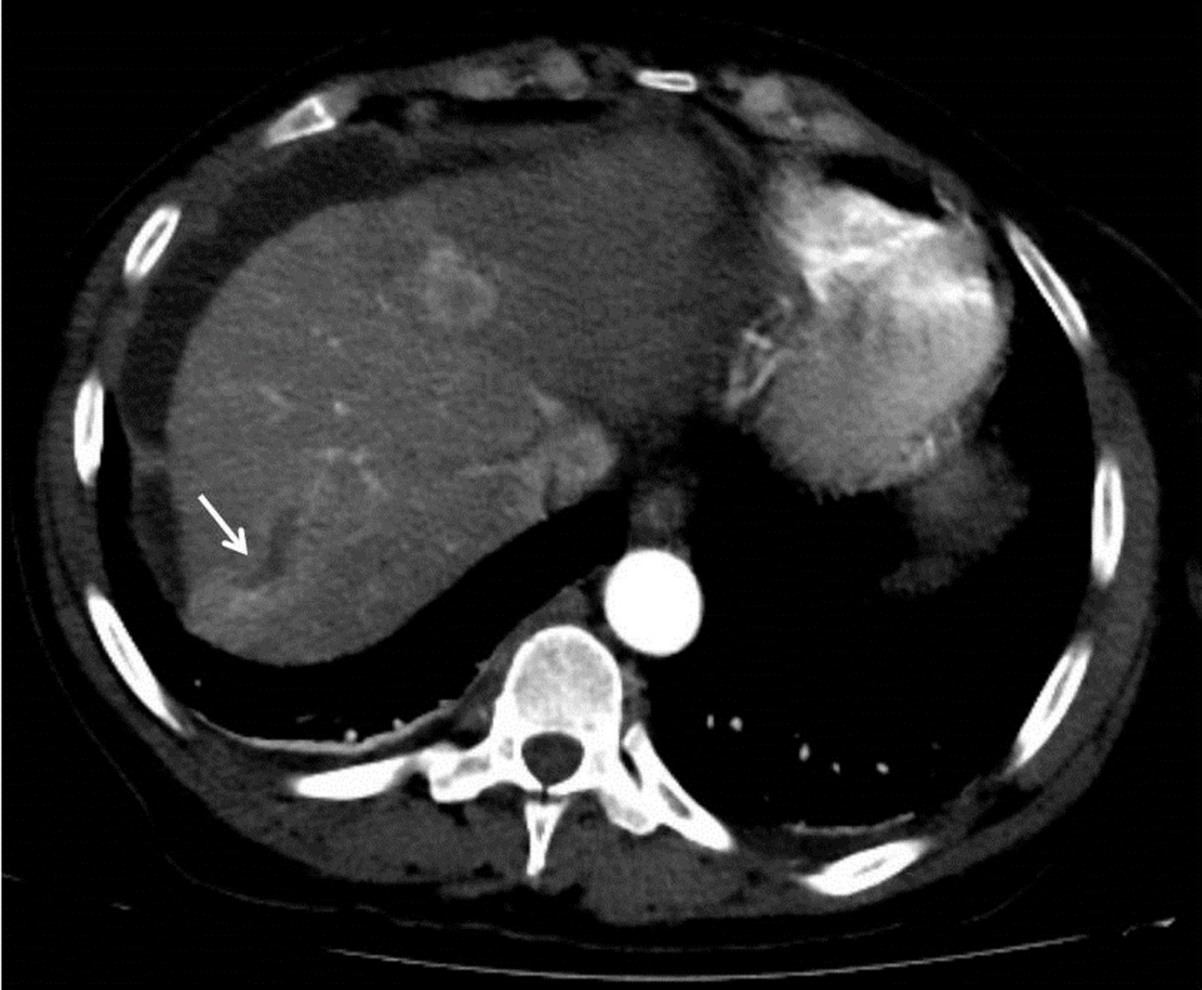
Computed tomography image of the liver with contrast showing about 4 x 2 cm-sized right main portal vein thrombosis (white thin arrow).

The patient’s alpha-fetoprotein and carbohydrate antigen 19-9 were both elevated at 76.5 ng/mL and 168.4 U/mL, respectively. HCC was diagnosed from the characteristic CT findings based on the diagnostic guideline of the 2016 American Association for the Study of Liver Diseases.^6^ A CT of the chest ruled out intrathoracic metastases and primary breast or lung cancer.

A work-up for hypercalcemia showed low serum intact parathyroid hormone (PTH) (14 pg/mL), low 25-hydroxyvitamin D (10 ng/mL), and normal 1,25-dihydroxyvitamin D (24 pg/mL). Bone-specific alkaline phosphatase was elevated at 25.1 ug/L. Urine protein electrophoresis showed no protein. PTHrP was found to be elevated at 39 pg/mL. HHM was diagnosed.

The patient was started on ergocalciferol (vitamin D2) 50,000 units weekly for a period of 12 weeks. Despite IV fluid administration, total serum calcium levels continued to remain high. Given her diagnosis of cirrhosis, IV hydration was discontinued to prevent fluid overload. She was given a dose of IV furosemide (a loop diuretic agent) 20 mg to which the total serum calcium did not respond. The patient was then started on IV zoledronic acid (a bisphosphonate that prevents bone resorption) 2 mg trial on day 9 after presentation with no improvement in calcium level.

The Tumor Board at the authors’ institution recommended systemic treatment options including targeted therapy or immunotherapy depending on performance status. Patient and the family declined definitive anti-HCC treatment, and requested discharge with preferring clinic-based follow-up, an appointment at which the patient failed to show.

Twenty-two days later, she was readmitted to the authors’ hospital with worsening symptoms of confusion, abdominal pain, and overall weakness. Her total serum calcium was 11.9 mg/dL. Zoledronic acid 2 mg IV was started. At 72 hours after administration, total calcium was 11.5 mg/dL. Five days later, the patient was again administered 4 mg of IV zoledronic acid with a post-24 hour total calcium of 11.7 mg/dL. Her glucose level oscillated between 50-90 mg/dL and was never above 100 mg/dL in comparison to her previous admission. Upon discharge, her total calcium remained elevated at 11.7 mg/dL. The patient’s family had planned for her to follow-up at the Endocrinology, Oncology, and Hepatology clinics but died before those appointments, 40 days after initial hospital presentation.

## DISCUSSION

Our case showed a patient with newly diagnosed hypercalcemia in the less common setting of HCC, and a fulminant course of hypercalcemia which failed to be controlled with IV fluids, furosemide and bisphosphonate therapy. Her risk factors for HCC had included a previous diagnosis of hepatitis C, without treatment, and alcohol abuse.

Of the three mechanisms that lead to malignant hypercalcemia,^1^ 85% of published cases have been caused by bone metastases whereas HHM was only found in approximately 10-15% of cases.^7^ It is unclear whether our patient had bony metastasis since a bone scan was not performed. We have concluded that a bone scan would have been unlikely to change the patient’s clinical outcome as her hypercalcemia was more likely secondary to PTHrP combined effects on her bones and kidneys. Other laboratory findings such as low intact PTH and normal 1,25-dihydroxyvitamin D with elevated PTHrP were also consistent with HHM.^8,9^

In 2005, Huh et al. reported a median survival time of 120 days after an HCC diagnosis, and 15 days after the development of HHM.^10^ This is consistent with our patient’s model for end-stage liver disease (MELD) score of 16 points with an estimated three-month mortality of 6%. In 2002, Luo et al. reported the median survival from the occurrence of paraneoplastic manifestation as 36 days.^11^ This data is also consistent with the 40-day survival of our patient.

Other well-recognized paraneoplastic factors associated with poor prognosis in HCC patients are hypoglycemia and hypercholesterolemia. Hypercholesterolemia is usually developed earlier in the course while hypoglycemia and hypercalcemia are considered terminal events.^5^ Our case also demonstrated episodes of hypoglycemia during her second hospitalization, perhaps due to her rapid tumor growth or inability of liver to meet metabolic demands.

Regarding the treatment of HCC, clinical evidence suggests that mass resection with or without lymph node dissection in patients with preserved hepatic function (Child-Pugh class A) is the first step.^6^ Our patient, however, demonstrated compromised liver function as evidenced by a Child-Pugh score of 11 points (class C) with a total bilirubin of 3.4 mg/dL, albumin of 1.94 g/dL, international normalized ratio of 2.18, lack of ascites, and the grade 2+ encephalopathy with behavioral changes. Furthermore, she did not meet the Milan or University of California San Francisco liver transplant criteria as she had an involvement of the right main portal vein and its proximal and anterior branches. The latter also made mass resection a less feasible option.

The response rate of HHM to IV bisphosphonates has been found to be about 57%.^12^ Oida et al. evaluated 17 cases of HCC-associated hypercalcemia and found a 100% response rate to IV bisphosphonates.^13^ However, published case reports of PTHrP- mediated hypercalcemia still show mixed outcomes in responsiveness to medical therapy, especially bisphosphonate therapy. Wimalawansa reported that serum PTHrP concentration >12 pg/mL is mostly associated with smaller reduction in hypercalcemia and with high recurrence within 14 days of therapy.^14^

As second-line options, calcitonin and glucocorticoid could have been considered. However, the medical team was concerned about calcitonin use given its possible association with malignancy,^15^ especially regarding liver cancer as recent population-based case control study showed that calcitonin use might increase the risk of liver cancer.^16^ Corticosteroid therapy was deferred given its limited effectiveness to 1,25-dihydroxyvitamin D-related hypercalcemia seen in granulomatous disease.^17^

Many of the case reports of HCC-associated hypercalcemia showed normalization of calcium level by transcatheter arterial embolization (TACE).^18–20^ However, we were not able to perform TACE because the patient had Child-Pugh class C with clinical hepatic encephalopathy and malignant portal vein thrombosis. These are all absolute contraindications for TACE.^20^ Mallik et al. observed that achieving a reduction in calcium levels could prolong patient survival.^12^ Our patient’s liver function continued to decline as demonstrated by the doubling of AST level over 30 days. Our patient’s worsening encephalopathy possibly further contributed to her non-adherence with laboratory and outpatient follow-up recommendations.

This case may have limited applicability to other patients. First, it was difficult for us to prove that the patient died directly from hypercalcemia as the cause of death could have been multifactorial. While it is possible that her hypoglycemia might have contributed to her death, it was noted that the glucose levels improved prior to the hospital discharge while the calcium levels were still elevated. Another critical point is that the patient’s post-discharge hypercalcemia was not properly controlled and monitored.

The zoledronic acid was started with 2 mg trials twice due to a concern for side effects even though 4 mg is the standard starting dose. While it is recommended to wait at least seven days after the zoledronic acid treatment to consider repeat treatment, post-treatment calcium level was obtained only 24 hours to three-to-four days in our case. In addition, patient’s calcium levels were not properly followed as she was discharged from the hospital per her request and she failed to go to outpatient appointments.

## CONCLUSIONS

This case report suggests that intensive medical therapy and monitoring with proper patient education should be considered when managing HHM secondary to HCC. This case also suggests the importance of a proper transition from inpatient setting to optimize clinic-based primary care physician follow-up.

Although HHM secondary to PTHrP production in HCC is rare, it is possible and should be considered on the differential diagnosis when presented with similar clinical scenario of hypercalcemia. Given that hypercalcemia can be a poor prognostic factor with possible fulminant and fatal clinical course, more aggressive medical therapy for hypercalcemia should be considered when a patient with HCC shows hypercalcemia. Furthermore, careful consideration of the feasibility of primary cancer treatment such as mass resection, TACE, and/or systemic therapy such as targeted therapy or immunotherapy may be critical in the management of HCC-associated HHM.

### Conflict of Interest

The authors declare no conflict of interest.
